# Analysis of Triple-Frequency PPP Ambiguity Resolution with Different Ambiguity Fixing Methods

**DOI:** 10.3390/s26206553

**Published:** 2026-10-15

**Authors:** Qiuyuan Yang, Guoqiang Jiao, Ke Su, Yuze Yang

**Affiliations:** 1 Shanghai Astronomical Observatory, Chinese Academy of Sciences, Shanghai 200030, China; a13417077863@163.com; 2 University of Chinese Academy of Sciences, Beijing 100049, China; 3 Beijing Institute of Tracking and Telecommunications Technology, Beijing 100094, China; jiaoguoqiang18@mails.ucas.ac.cn; 4 National Key Laboratory of Intelligent Spatial Information, Beijing 100094, China; suke17@mails.ucas.ac.cn; 5 Space Engineering University, Beijing 101416, China

**Keywords:** precise point positioning, ambiguity resolution, triple-frequency GNSS, Partial Ambiguity Resolution, Best Integer Equivariant, Integer Aperture Bootstrapping

## Abstract

Accurate ambiguity resolution (AR) is critical for precise point positioning (PPP). Triple-frequency observations enable longer wavelength combinations, improving AR performance. This study evaluates four AR algorithms, Integer Least Squares (ILS), Partial Ambiguity Resolution (PAR), Best Integer Equivariants (BIEs), and Integer Aperture Bootstrapping (IAB), using triple-frequency observations from 275 MGEX stations and a kinematic vehicle. The results show that PAR achieves the fastest convergence, within 1.0 min at the 68th percentile and 3.0 min at the 84th percentile. Satellites excluded by PAR are typically 10–12° lower in elevation than retained ones, and their exclusion is driven more by ambiguity geometry and noise than by large fractional parts. In the kinematic vehicle test, BIE offers superior stability, with RMS errors of 0.042 m (east) and 0.059 m (north), while the ILS fixing rate drops below 6%, and PAR remains at 97%. A dual-frequency baseline further shows that the third frequency benefits all four methods: in terms of the station-averaged 86th-percentile convergence time, ILS and IAB gain the most (−43% and −42% horizontally), whereas its effect on positioning precision is modest (1–28% in all components), with PAR remaining the most precise method.

## 1. Introduction

Precise point positioning ambiguity resolution (PPP-AR) is a key technique for high-precision global navigation satellite system (GNSS) observations. It has been shown since the pioneering work of Ge et al. [1] that PPP-AR reduces the convergence time from about 30 min to a few minutes and improves the positioning accuracy of PPP. More recent studies further demonstrate that PPP-AR can extract real-time multi-GNSS ionospheric delays [2] and tropospheric delays [3], which are invaluable for studying Earth’s atmosphere, geodynamics, space weather, and physics [4]. In addition, PPP-AR also enhances stability and frequency stability while reducing the noise level of time transfer [5].

However, unlike Real-Time Kinematic (RTK), PPP-AR cannot form double-differenced observations to eliminate hardware-dependent phase biases. Therefore, it is necessary to estimate and correct satellite-specific uncalibrated phase delays (UPDs) [6]. Different algorithms can be employed to fix ambiguities to their correct integer values once the UPDs are corrected. AR methods can be classified into three categories, following the nested relationship I⊂IA⊂IE: integer estimators (I-class) [7], integer aperture estimators (IA-class), and integer equivariant estimators (IE-class) [8]. The I-class requires the pull-in regions to be seamless, non-overlapping, and invariant under integer translations; the IA-class allows for apertured regions with gaps; and the IE-class further permits overlaps while still maintaining the property of integer removal and restoration [9]. Within the IE-class, the Best Integer Equivariant (BIE) estimator was first introduced for precise point positioning by Wen et al. [10] using simulated observations. Subsequently, Odolinski and Teunissen [11] provided the first comprehensive performance analysis of BIE using real multi-GNSS data collected by low-cost, single- and dual-frequency receivers for short- to long-baseline RTK positioning, demonstrating its minimum-MSE optimality in practice. The Least-squares AMBiguity Decorrelation Adjustment (LAMBDA) 4.0 toolbox [12] has further demonstrated that different ambiguity resolution methods yield distinct statistical and computational characteristics.

Triple-frequency signals substantially enhance PPP performance through improved ambiguity resolution. In the multi-constellation PPP context, this benefit was demonstrated early by Laurichesse and Blot [13], who showed that extra-wide-lane and wide-lane-only phase combinations enable quasi-instantaneous convergence. Subsequent studies have consistently confirmed these benefits, J. Geng et al. [14] injected uncombined triple-frequency GPS/BeiDou/Galileo/QZSS data into PPP and showed that triple-frequency PPP-AR improved 10 min positioning accuracy by about 50%, increased the 2 min initialization fixing rate from 26% to 48%, and reduced the average initialization time from 9 to 6 min. M. A. Rabbou et al. [15] demonstrated that, in both static and kinematic conditions, triple-frequency GPS/Galileo PPP models consistently outperformed the dual-frequency PPP model, with the semi-decoupled model achieving the most substantial improvements. Gazzino and Lelarge [16] proposed a cascading EWL–WL ambiguity resolution scheme exploiting up to four frequencies, achieving instantaneous extra-wide-lane fixation and very fast wide-lane fixing. T. Geng et al. [17] confirmed that OSB products support over 95% wide-lane fixing rates and reduce triple-frequency convergence time by 43–50%, although performance remains receiver-dependent and benefits saturate beyond three frequencies owing to antenna calibration limitations.

Further advances in modeling have also contributed to these improvements. Xiao et al. [18] introduced a unified uncombined observation model that improved accuracy by 49–60% horizontally and 21–30% vertically for BDS and Galileo, highlighting the consistent advantage of triple-frequency signals over float solutions. Liu et al. [19] enhanced real-time instantaneous decimeter-level PPP ambiguity resolution by integrating the Galileo E6 signal, achieving accuracies of 0.13 m (east), 0.13 m (north), and 0.57 m (up), outperforming the conventional E1/E5a/E5b combination. Zhang et al. [20] demonstrated that adding more frequency observations enhances integer PPP (IPPP) time and frequency transfer performance. Specifically, triple-frequency IPPP improved zero-baseline time transfer accuracy beyond the dual-frequency solution, while quad-frequency IPPP achieved the best long-term frequency stability.

Although triple-frequency PPP-AR has achieved substantial progress, several practical challenges remain unresolved. Existing studies primarily focus on demonstrating the benefits of additional frequencies or introducing new bias-correction products, but limited attention has been paid to how different ambiguity resolution strategies behave under complex conditions. Most operational implementations, including widely used open-source PPP-AR packages such as PRIDE-PPPAR [21] and GREAT-PVT [22], default to employing a single ILS method, even though ILS, PAR, BIE, and IAB differ fundamentally in terms of optimality criteria, robustness, and computational complexity. Consequently, it remains unclear how these methods compare when applied to triple-frequency PPP-AR, where cycle slips, multipath, urban masking, and dynamic motion may influence their stability and fixing reliability. To address these issues, this study adopts a comparative and experiment-driven approach. We focus on characterizing the practical behavior of representative estimators from the I-class, IA-class, and IE-class and evaluating how their theoretical properties translate into real PPP performance. We shall design test scenarios encompassing static and dynamic conditions as well as diverse observation environments, analyzing differences among methods in convergence time, positioning accuracy, fixing rate, and computational efficiency. It is anticipated that this research will clarify the applicable boundaries and comparative advantages of different fixed ambiguity algorithms in triple-frequency PPP, thereby providing concrete data support and a decision-making basis for algorithm selection and software optimization in high-precision PPP applications within complex scenarios.

## 2. PPP Ambiguity Resolution

### 2.1. PPP Model for Triple-Frequency Raw Observation

Generally, the uncombined GNSS pseudorange and carrier phase raw observation equations can be expressed as follows [23]:

(1) $$\left\{\begin{array}{c} P_{r,j}^{s}=\rho_{r}^{s}+dt_{r}-dt^{s}+T_{r}^{s}+\mu_{j}\cdot I_{r,1}^{s}+d_{r,j}+d_{j}^{s}+\epsilon_{r,j}^{s}\\ L_{r,j}^{s}=\rho_{r}^{s}+dt_{r}-dt^{s}+T_{r}^{s}-\mu_{j}\cdot I_{r,1}^{s}+\lambda_{j}\cdot N_{r,j}^{s}+\varphi_{r,j}+\varphi_{j}^{s}+\xi_{r,j}^{s} \end{array}\right.$$

where *s*, *r* and *j*(*j* = 1,2,3) respectively denote satellite, receiver and carrier phase frequency. $\rho_{r}^{s}$ is the geometric distance between satellites *s* and receivers *r*. $P_{r,j}^{s}$ and $L_{r,j}^{s}$ represent the raw code and carrier phase observations for frequency *j* in meters. *dt_r_* and *dt^s^* are the clock bias of receivers and satellites in meters, with the light speed absorbed. $T_{r}^{s}$ denotes the tropospheric delay in the direction of line of sight (LOS). $I_{r,1}^{s}$ is the ionospheric oblique delay at the first frequency. $\mu_{j}=f_{1}^{2}/f_{j}^{2}$ is the ionospheric amplification factor, and *f_j_* denotes the carrier phase frequency *j*. *λ_j_* and $N_{r,j}^{s}$ are the wavelength and ambiguity of the carrier phase. *d_r_*_,*j*_ and $d_{j}^{s}$ are the pseudorange hardware bias of receivers and satellites, and *φ_r_*_,*j*_ and $\varphi_{j}^{s}$ accordingly denote the carrier phase bias of receivers and satellites. $\epsilon_{r,j}^{s}$ and $\xi_{r,j}^{s}$ respectively denote the unmodeled combined errors such as observation noise and multipath effects corresponding to the pseudorange and carrier phase observation.

Consequently, ambiguity fixing in triple-frequency PPP generally uses a cascading strategy with linearly independent ambiguity combinations, the extra-wide-lane (EWL), wide-lane (WL), and narrow-lane (N1) combinations, to progressively enhance the reliability of integer resolution [24]. The corresponding ambiguity can be formed with the individual ambiguities at three frequencies and are expressed as follows:

(2) $$\left[\begin{array}{c} {\hat{N}}_{\mathrm{ewl},r}^{s}\\ {\hat{N}}_{\mathrm{wl},r}^{s}\\ {\hat{N}}_{1,r}^{s} \end{array}\right]=\left[\begin{array}{ccc} 0 & 1 & -1\\ 1 & -1 & 0\\ 1 & 0 & 0 \end{array}\right]\left[\begin{array}{c} {\hat{N}}_{1,r}^{s}\\ {\hat{N}}_{2,r}^{s}\\ {\hat{N}}_{3,r}^{s} \end{array}\right]$$

The EWL combination possesses the longest wavelength, and its float uncertainty in cycles therefore remains well below 0.25 cycles. Consequently, EWL ambiguity can be directly rounded to its nearest integer value [25]:

(3) $${\overset{ˇ}{N}}_{\mathrm{ewl},r}^{s}=\mathrm{round}\left({\hat{N}}_{\mathrm{ewl},r}^{s}\right)$$

The fixed EWL ambiguities are then introduced as known constraints to enhance the precision of the subsequent WL and N1 ambiguity estimation. The following section describes the candidate estimators for fixing WL and N1 ambiguities.

### 2.2. Triple-Frequency Ambiguity Resolution

#### 2.2.1. Integer Least-Squares Estimator

**Figure 1:**
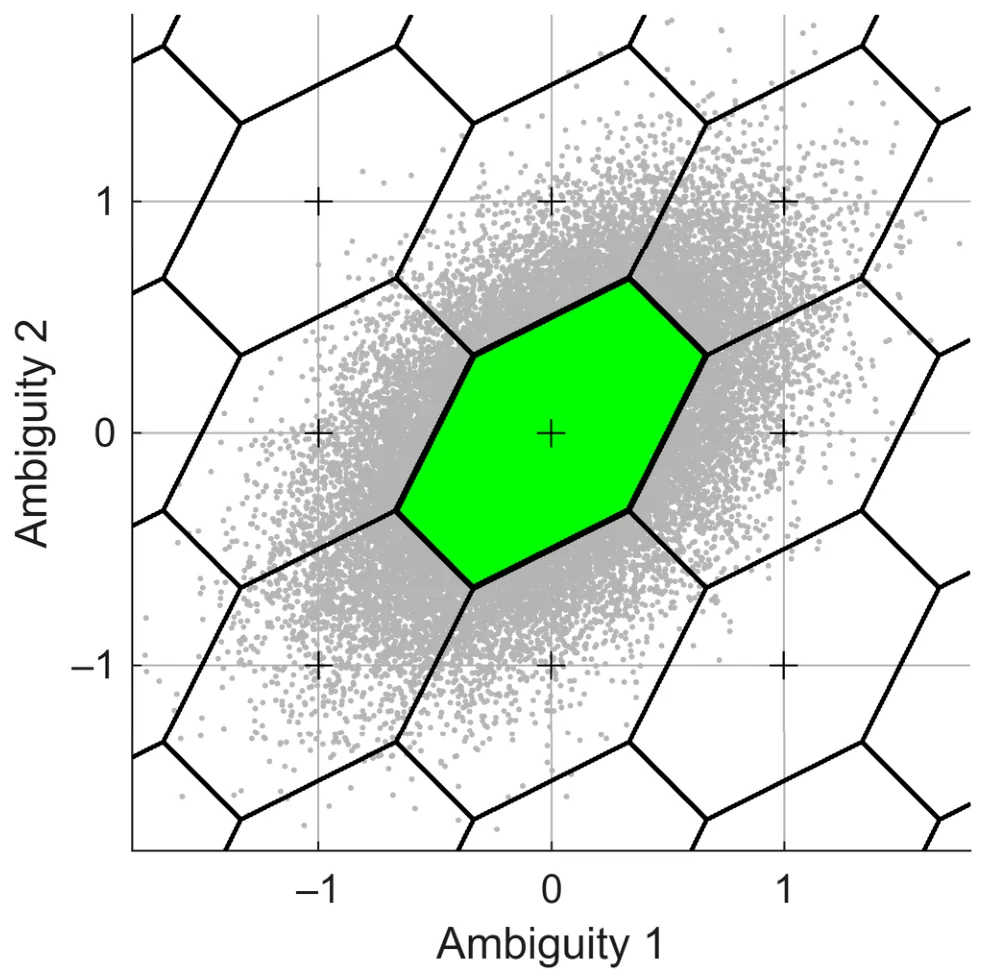
An illustration of the ILS pull-in region with the float ambiguity samples pulled to the true integer vector (i.e., *a* = 0) rendered in green, while the grey points are the ambiguities that are not pulled in.

The ILS estimator is optimal within the class of integer estimators [26,27]. This optimality is defined by maximizing the ambiguity resolution success rate. Let $\hat{a}\in R^{n}$ denote the float estimator, with *Q*∈ℝ*^n^* ^× *n*^ being its symmetric positive-definite variance–covariance (VC) matrix, where *n* is the number of ambiguities to be fixed. The ILS estimate is obtained by solving the following integer minimization problem:

(4) $${\overset{ˇ}{a}}_{\mathrm{ILS}}=\arg\underset{z\in Z^{n}}{\min}\;{∥\hat{a}-z∥}_{Q_{\hat{\alpha }\hat{\alpha }}}^{2}$$

with ${||x||}_{Q}^{2}\overset{\mathrm{def}}{=}x^{T}Q^{-1}x$ being the vector norm weighted by matrix *Q*. We define the pull-in region as a subset ***S_z_*** that contains all real-valued vectors that will be mapped by ***S*** to the same integer vector ***z***∈ℤ. In this case, the pull-in region of the ILS estimator can be given as:

(5) $$P_{z,\mathrm{ILS}}=\left\{x\in R^{n}|\left|\frac{u^{T}Q_{\hat{\alpha }\hat{\alpha }}^{-1}\left(x-z\right)}{∥u{∥}_{Q_{\hat{\alpha }\hat{\alpha }}}}\right|\leq \frac{1}{2}∥u{∥}_{Q_{\hat{\alpha }\hat{\alpha }}},\;\forall u\in Z^{n}\right\},\;z\in Z^{n}$$

where $\frac{u^{T}Q_{\hat{\alpha }\hat{\alpha }}^{-1}\left(x-z\right)}{∥u{∥}_{Q_{\hat{\alpha }\hat{\alpha }}}}$ is the signed length of the orthogonal projection of (***x*** − ***z***) onto the direction ***u***. Each ***u*** therefore defines a band, perpendicular to ***u*** and of half-width $\frac{1}{2}{∥u∥}_{Q_{\hat{\alpha }\hat{\alpha }}}$, centered at *z*; the ILS pull-in region is hence the intersection of these banded subsets. An example of a 2D pull-in region is given in Figure 1, with the ILS regions being hexagons that seem to better fit the distribution of the float ambiguity estimates.

ILS fixing is designed to resolve all integer ambiguities. However, in practice, it is often not feasible to reliably fix every ambiguity. In such situations, only a subset of ambiguities can be fixed with confidence, giving rise to the concept of PAR [28,29]. It should be emphasized that PAR is not an estimator distinct from ILS; it applies the same ILS integer estimator to a subset of ambiguities selected by a ratio-test-driven elimination rule. Thus, when the full-set ILS fails the ratio test, PAR iteratively removes unreliable ambiguities and fixes the largest subset that passes statistical validation. A critical aspect of this approach is the selection of which ambiguities to fix. In this work, ambiguities associated with weak ratio test values are progressively excluded until the remaining set satisfies statistical validation criteria. The flowcharts of the algorithm are presented in the following pseudocode.

In Algorithm 1, *ρ_min_* and *s_min_* represent the minimum ratio threshold set by the user to accept the integer solution and the minimum number of satellites needed for positioning, respectively. $\mathrm{ILS}(\hat{a},Q_{\hat{\alpha }\hat{\alpha }})$ denotes the ILS search, which returns the fixed ambiguity vector $\overset{ˇ}{a}$ together with the two smallest quadratic forms $\overset{ˇ}{s}\left[0\right]\leq \overset{ˇ}{s}\left[1\right]$ of residuals ${\left\|\overset{ˇ}{a}-\hat{a}\right\|}_{Q_{\hat{\alpha }\hat{\alpha }}}^{2}$, from which the ratio test statistic is formed as $\rho_{\mathrm{test}}=\frac{\overset{ˇ}{s}\left[1\right]}{\overset{ˇ}{s}\left[0\right]}$. The threshold *ρ_min_* is the minimum acceptable ratio value, set to 1.8 in this study. The core function $\mathrm{Remove}\mathrm{Ambiguity}(\hat{a},Q_{\hat{\alpha }\hat{\alpha }},j)$ removes the *j*-th entry of $\hat{a}$ together with the corresponding row and column of $Q_{\hat{\alpha }\hat{\alpha }}$.
**Algorithm 1:** Partial Ambiguity Resolution**Input:** $\hat{a}\in R^{n\times 1},Q_{\hat{\alpha }\hat{\alpha }}\in R_{+}^{n\times n},\rho_{\min},s_{\min}$ **Output:** $\overset{ˇ}{a}\in Z^{m\times 1},\rho_{\mathrm{test}}$**for** *i* = *n*:*s_min_* **do**     $\left(\overset{ˇ}{a},\;\overset{ˇ}{s}\right)=\mathrm{ILS}(\hat{a},Q_{\hat{\alpha }\hat{\alpha }})$     $\rho_{\mathrm{test}}=\frac{\overset{ˇ}{s}\left[1\right]}{\overset{ˇ}{s}\left[0\right]}$     **if** *ρ_test_* > *ρ_min_*         **return** $\left(\overset{ˇ}{a},\rho_{\mathrm{test}}\right)$     **for** *j* = 1:*i* **do**         ${\hat{a}}_{j},Q_{j}=\mathrm{Remove Ambiguity}(\hat{a},Q_{\hat{\alpha }\hat{\alpha }},j)$         $\left(\cdot ,{\overset{ˇ}{s}}_{j}\right)=\mathrm{ILS}({\hat{a}}_{j},Q_{j})$         $\rho [j]=\frac{\overset{ˇ}{s}\left[1\right]}{\overset{ˇ}{s}\left[0\right]}$     $\left(\hat{a},Q_{\hat{\alpha }\hat{\alpha }}\right)$ **=** $\mathrm{Remove Ambiguity}(\hat{a},Q_{\hat{\alpha }\hat{\alpha }},\max\left(\rho \right))$

#### 2.2.2. Best Integer Equivariant Estimator

The optimality criterion for the BIE estimator is the minimum Mean Squared Error (MSE). The MSE is a widely used probabilistic measure of an estimator’s closeness to its target value and is often employed to assess the quality of the float solution itself. The BIE estimator is given by [30]:

(6) $$\begin{array}{c} {\overset{˘}{a}}_{\mathrm{BIE}}=\frac{\sum_{z\in Z^{n}}\;z\exp\left(-\frac{∥\hat{a}-z{∥}_{Q_{\hat{a}\hat{a}}}^{2}}{2}\right)}{\sum_{u\in Z^{n}}\;\exp\left(-\frac{∥\hat{a}-u{∥}_{Q_{\hat{a}\hat{a}}}^{2}}{2}\right)} \end{array}$$

**Figure 2:**
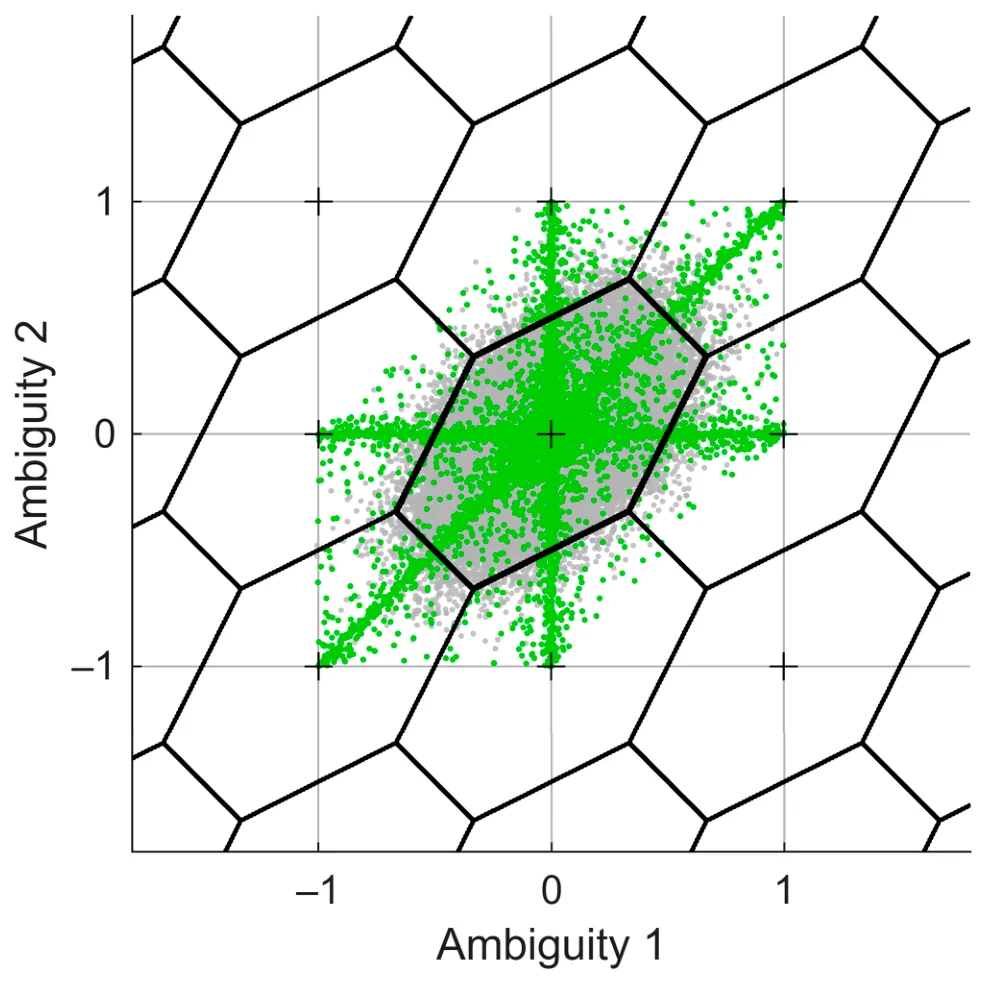
An illustration of the BIE solution (in green) and float solution (in gray) for 40,000 samples. Being minimum-MSE-optimal, the BIE estimator achieves notable performance improvements in PPP-AR applications [31]. In terms of the MSE, BIE solutions consistently outperform both float and fixed solutions.

BIE does not return an integer-fixed ambiguity vector; instead, it computes an optimal equivariant estimator that smooths candidate integer vectors. A simulation of BIE solutions is given in Figure 2, where the BIE solutions exhibit a characteristic star-shaped distribution, which violates both the ‘no gaps’ and ‘no overlaps’ conditions required for integer aperture estimators.

#### 2.2.3. Integer Aperture Bootstrapping Estimator

The IAB estimator [32] extends the Integer Bootstrapping (IB) estimator by introducing a controllable failure rate. The IB estimator can be seen as a generalization of the process of integer rounding, but it also takes some of the correlation between the ambiguities into account. The IB estimator fixes the ambiguities sequentially: each ambiguity is rounded to the nearest integer conditioned on the previously rounded ambiguities, and the entries of the bootstrapped estimator are given as:

(7) $$\left\{\begin{array}{c} {\overset{ˇ}{a}}_{B,1}=\left[{\hat{a}}_{1}\right]\\ {\overset{ˇ}{a}}_{B,2}=\left[{\hat{a}}_{2|1}\right]=\left[{\hat{a}}_{2}-\sigma_{21}\sigma_{1}^{-2}\left({\hat{a}}_{1}-{\overset{ˇ}{a}}_{B,1}\right)\right]\\ \vdots \\ {\overset{ˇ}{a}}_{B,n}=\left[{\hat{a}}_{n|N}\right]=\left[{\hat{a}}_{n}-\sum_{j=1}^{n-1}\sigma_{n,j|J}\sigma_{j|J}^{-2}\left({\hat{a}}_{j|J}-{\overset{ˇ}{a}}_{B,J}\right)\right] \end{array}\right.$$

where [⋅] denotes rounding to the nearest integer, *σ_i_*_,*j*|*J*_ denotes the covariance between ${\hat{a}}_{i}$ and ${\hat{a}}_{j|J}$, and $\sigma_{j|J}^{2}$ is the variance of ${\hat{a}}_{j|J}$. The shorthand notation ${\hat{a}}_{i|I}$ stands for the *i*th least-squares ambiguity obtained through a conditioning on the previous *I* = {1,…,(*i* − 1)} sequentially rounded ambiguities. The pull-in regions of the IB estimator can be defined as:

(8) $$P_{z,\mathrm{IB}}=\left\{x\in R^{n}|\left|c_{i}^{T}L^{-1}(x-z)\right|\leq \frac{1}{2},i=1,\ldots ,n\right\},\forall z\in Z^{n}$$

with ***c_i_*** being the canonical unit vector with ‘1’ as its *i*-th entry, ‘0’ otherwise. The matrix *L* is the lower unit-triangular matrix obtained in the L^T^DL-decomposition.

The IAB estimator uses an aperture parameter *β***_IAB_**∈[0, 1] to scale the IB pull-in region, allowing users to explicitly control the failure rate. The pull-in of the IAB estimator can be expressed as:

𝒫*_z_*_,IAB_ = *β*𝒫*_z_*_,IB_ (9)

**Figure 3:**
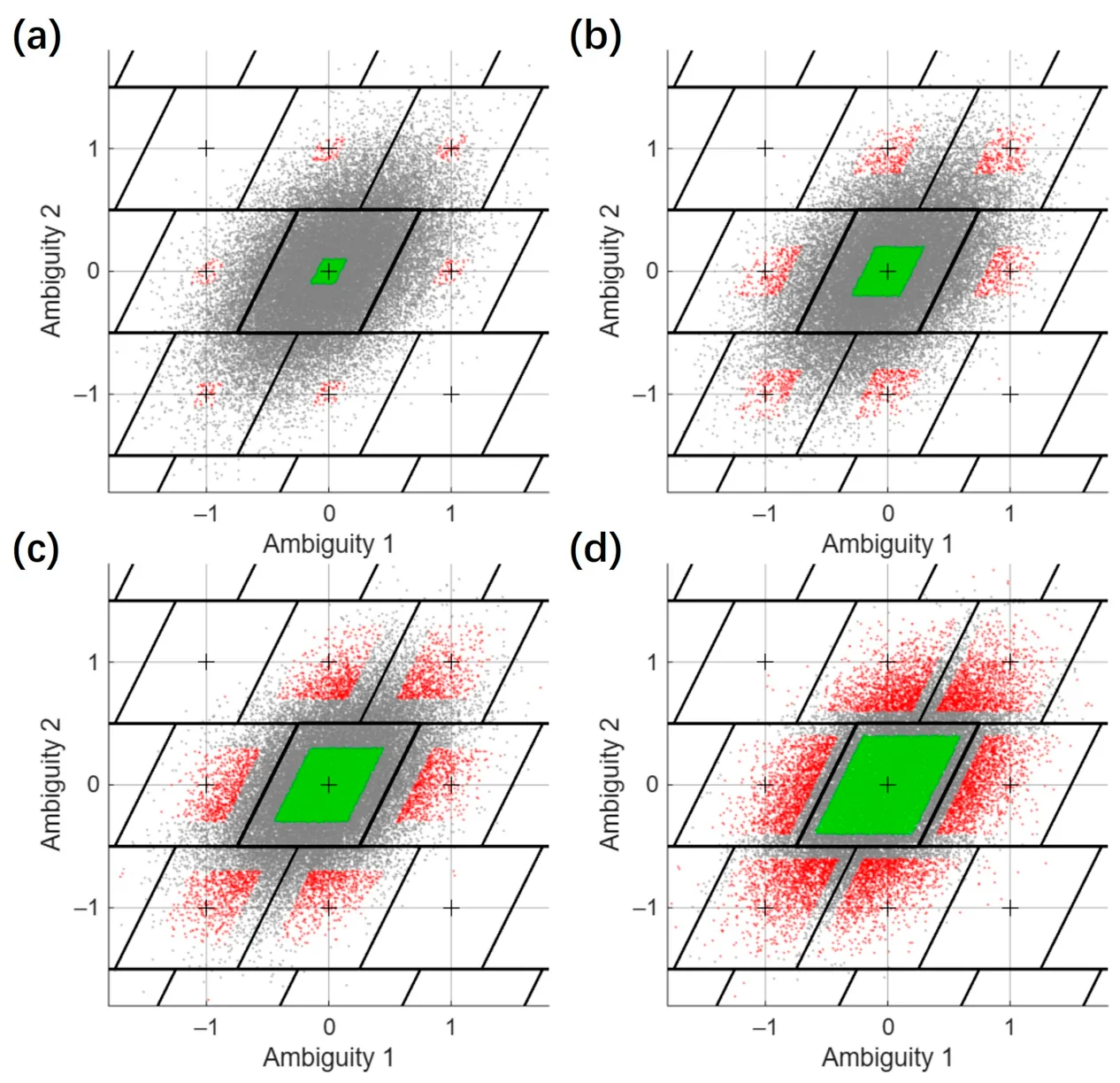
An illustration of the impact of the aperture parameter *β***_IAB_** values of 0.2, 0.4, 0.6 and 0.8 in subfigures (**a**–**d**). The outcomes are color-coded as follows: green, success; gray, failure; and red, incorrect. The results reveal a trade-off where a smaller *β***_IAB_** reduces the fixing rate, while a larger *β***_IAB_** increases the incorrect fixation rate.

In Figure 3, we illustrate the impact of different aperture parameters, i.e., *β***_IAB_** = [0.2, 0.4, 0.6, 0.8], where the IB pull-in regions are drawn as a reference.

## 3. Data Description and Processing Strategy

**Figure 4:**
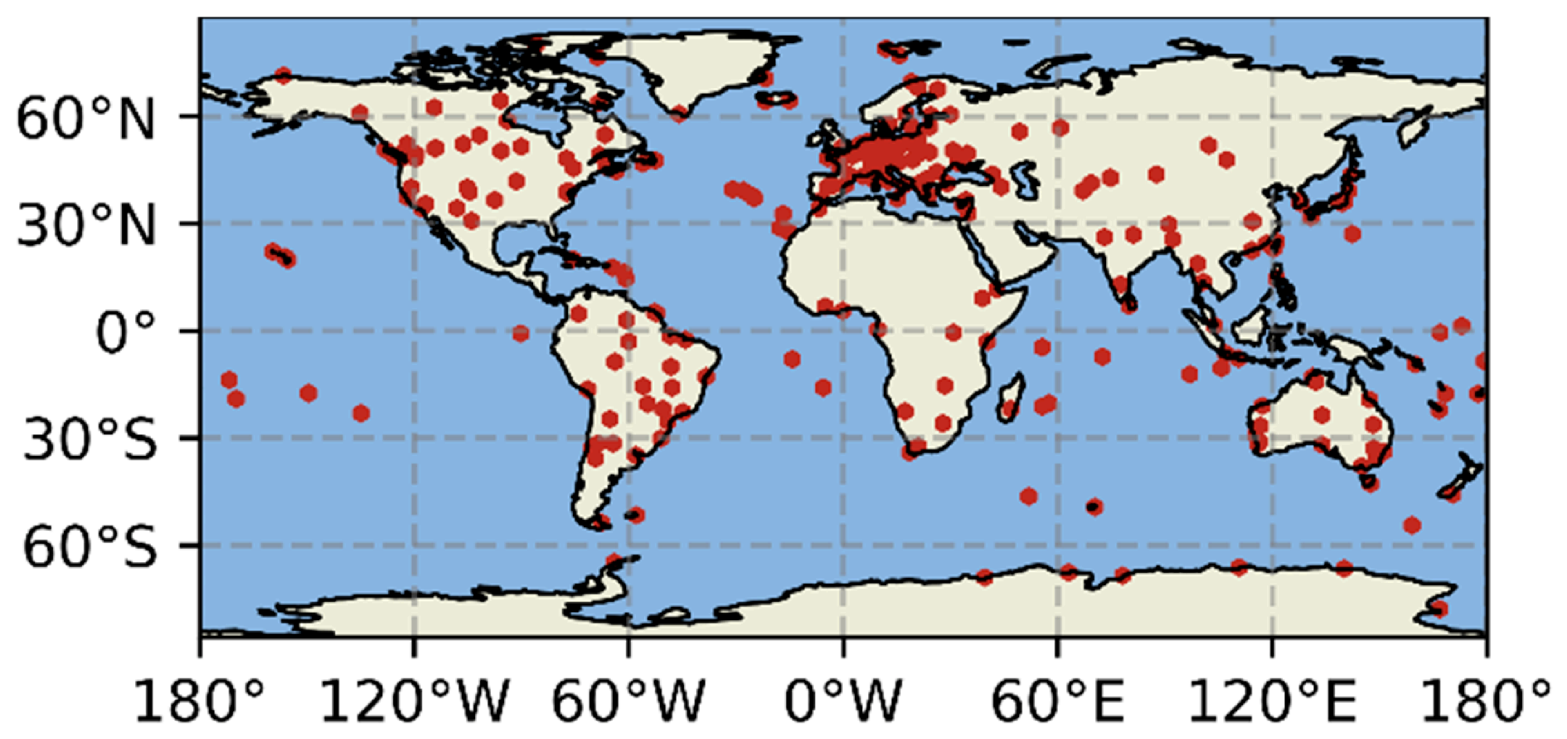
The geographical distribution of the selected 275 MGEX tracking stations.

In this section, we set up an experiment based on observation data collected from globally distributed IGS Multi-GNSS Experiment (MGEX) tracking stations. The distribution of these stations is illustrated in Figure 4, and we provide details on the data processing strategy we employed.

### 3.1. Dataset

To evaluate the performance of different AR methods in triple-frequency PPP fixed solutions, triple-frequency observations from 275 MGEX stations equipped with triple-frequency capable receivers were processed. The dataset spans 7 days (DOY 147–153) in May 2025, with a sampling interval of 30 s. the observations include GPS L1/L2/L5, Galileo E1/E5a/E6, and BDS B1I/B2a/B3I signals. The geographic distribution of the selected stations is shown in Figure 4.

### 3.2. Processing Strategy

To comprehensively evaluate the contribution of the triple-frequency PPP model and the AR methods, we compared the positioning performance of different AR methods under triple-frequency observations. The processing utilized post-processed satellite orbits and integer carrier phase clock products from the CODE, along with observable-specific signal bias products provided by Wuhan University. A simulated kinematic PPP mode was adopted, estimating station coordinates and receiver clock errors as white-noise processes. The float ambiguities were resolved and held constant over continuous arc segments while being reinitialized following any cycle slip occurrences. The detailed strategy for PPP-AR is summarized in Table 1. To isolate the contribution of the third frequency, the same datasets were additionally processed in dual-frequency mode (GPS L1/L2, Galileo E1/E5a and BDS B1I/B3I) with the WL/N1 cascading strategy, keeping all other settings unchanged.

Specifically, the ILS and IAB methods were implemented using the default configuration of the LAMBDA 4.0 toolbox, where the ratio test value for the ILS and PAR methods was set to 1.8, and the aperture parameter for the IAB method was set to 0.5. For the BIE method, the number of pre-given integer candidates was set to 200, following the strategy suggested in previous studies, which balances computational efficiency and estimation performance.

## 4. Results and Validation

This section first analyzes the computational time of different AR methods, alongside their relationship between time to first fix (TTFF) and convergence time, as well as the fixing rate and positioning precision. Subsequently, post-processing products and vehicle-collected data were used to validate the impact of different AR methods in terms of convergence speed and position accuracy.

### 4.1. Processing Time

The computational efficiency of AR techniques is critical for the practical deployment of real-time PPP-AR, particularly in high-rate environments where the dimensionality of the ambiguity vector can be large. To examine the algorithmic behavior in a controlled setting, a comprehensive instantaneous simulation was performed using 4000 randomly generated ambiguity vectors with dimensions spanning from 10 to 25. For each realization, synthetic float ambiguities and their corresponding variance–covariance matrices were generated, and the computational time of the four AR methods was evaluated. All computations were carried out on a desktop computer equipped with a 2.50 GHz processor, with the entire search procedure implemented in optimized C++.

Table 2 summarizes the mean computational time, standard deviation (STD), and average dimensional growth rate for the four AR methods. The IAB and ILS algorithms demonstrate the lowest computational burden, with average runtimes below 5 ms and modest growth rates (15.6% and 20.3%, respectively) as the ambiguity dimension increases from 10 to 25. This behavior reflects the compact search-and-shrink procedure of the LAMBDA method, which benefits from effective decorrelation and typically exhibits near linear scaling with decorrelated ambiguity vectors.

**Figure 5:**
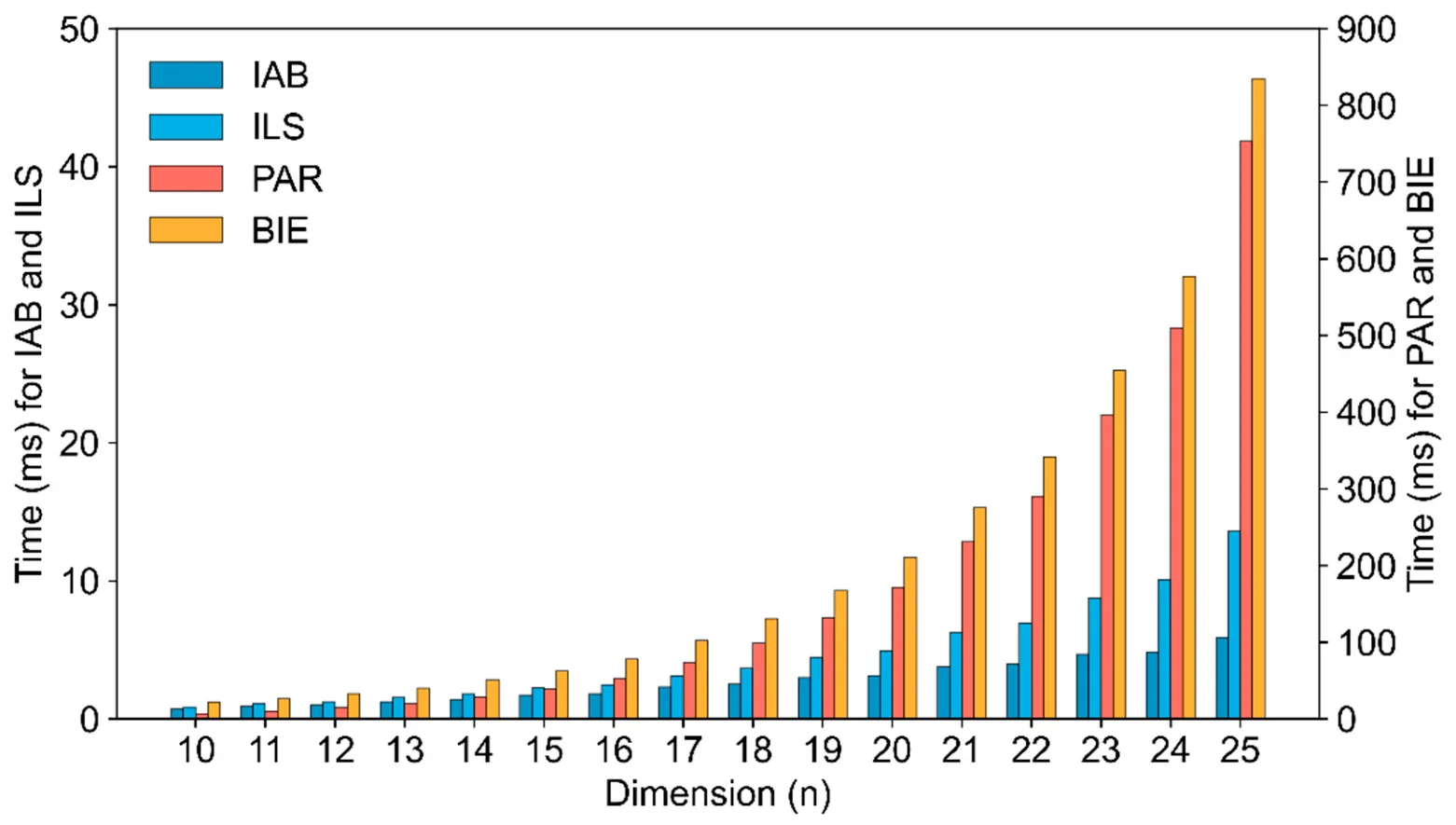
The average computational cost of IAB, ILS, PAR, and BIE as a function of ambiguity dimension.

Conversely, the PAR and BIE methods incur significantly higher computational loads and variability. The BIE algorithm exhibits a growth rate of 27.6%, which might result from its weighting strategy, in which each of the *K* integer candidates require a matrix–vector evaluation, resulting in O(*K*⋅*n*^2^) complexity per epoch, where *n* is the ambiguity dimension. The PAR method exhibits the steepest growth rate, which is mainly attributed to its iterative elimination strategy. In each iteration, the ambiguity with the smallest ratio value is selected and removed, and the remaining ambiguities must be re-estimated through a new ILS search with reduced dimension. When the ambiguity dimension becomes larger, this elimination–recomputation cycle occurs more frequently, and each iteration becomes more expensive, as shown in Figure 5.

The near-mean standard deviation of the PAR runtime reflects the strongly data-dependent nature of its iterative elimination process. When the initial ratio test is passed immediately, the cost reduces to that of a single ILS search. In contrast, epochs with weak float ambiguity precision require repeated removal and re-search cycles, each triggering additional ILS searches whose tree complexity varies with the conditioning of the decorrelated ambiguity. The runtime therefore varies by roughly an order of magnitude between the best and worst cases, yielding a heavy-tailed distribution. BIE shows similar dispersion (its standard deviation also exceeds the mean) because enumerating the pre-given 200 candidates becomes more expensive when the float ambiguity distribution is more dispersed.

### 4.2. Precise Point Positioning—Ambiguity Resolution Performance

#### 4.2.1. Fixed Capability

In this section, we evaluate the fixing capability of the three integer-commitment ambiguity resolution methods: PAR, ILS, and IAB. The BIE method is excluded from this comparison because it does not commit to a single integer solution but instead produces a weighted average of multiple integer candidates.

**Figure 6:**
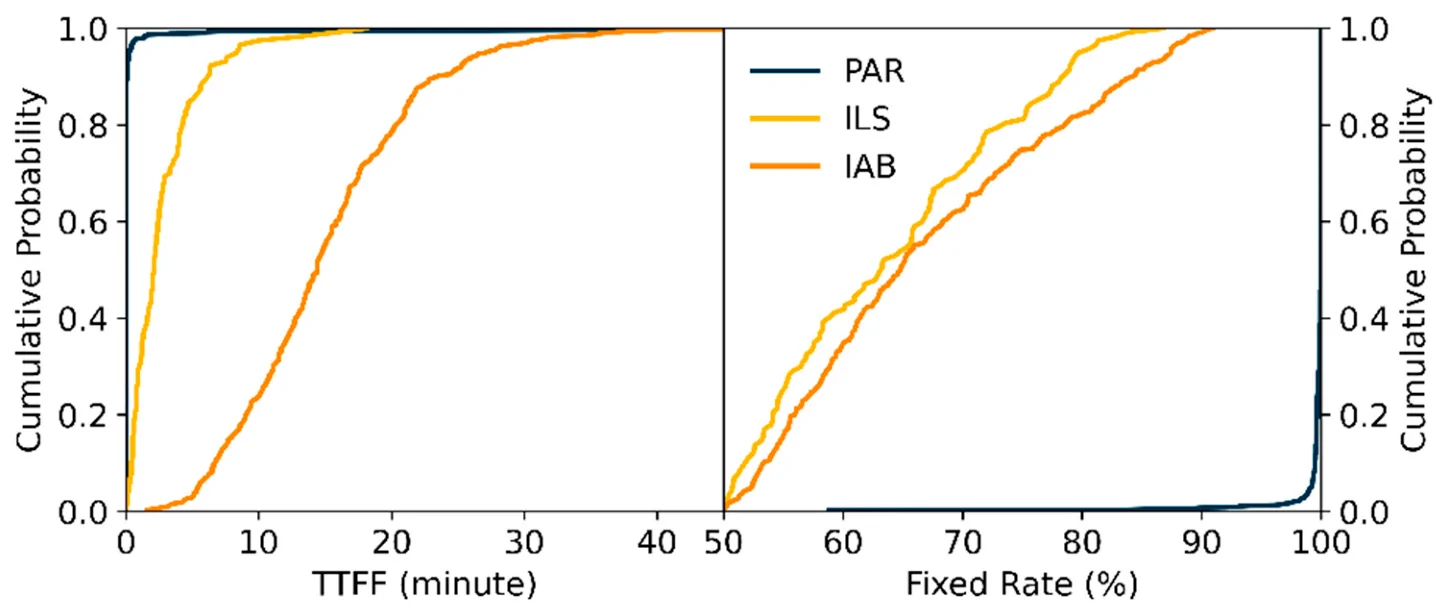
Cumulative distribution functions of TTFF (**left**) and fixing rate (**right**) for PAR, ILS, and IAB methods.

Figure 6 presents the cumulative distribution functions (CDFs) of TTFF (left panel) and the fixing rate (right panel). Here, the fixing rate is defined as the fraction of epochs in which an integer solution is accepted by the ratio test. It measures fixing availability and does not by itself certify the correctness of the accepted fix. With PAR, the 90th percentile of TTFF is approximately 1.5 min, and the 95th percentile remains below 3 min, confirming rapid convergence in the vast majority of sessions. The fixing rate distribution is equally remarkable: the median fixing rate exceeds 99.8%, and even the 5th percentile remains above 97%, indicating exceptional robustness across geographically diverse and ionospherically challenging stations. In contrast, the ILS estimator exhibits a 90th-percentile TTFF of approximately 13 min and a 95% level TTFF exceeding 20 min. Its fixing rate distribution is markedly inferior, with the 5th percentile falling below 80%. The IAB method performs the worst among the three, showing a 90th-percentile TTFF of approximately 18 min and a 5th-percentile fixing rate below 70%. These results indicate that the PAR method delivers substantially faster first-fix times and significantly higher fixing rates than the classical ILS and IAB methods.

#### 4.2.2. Convergence Time

**Figure 7:**
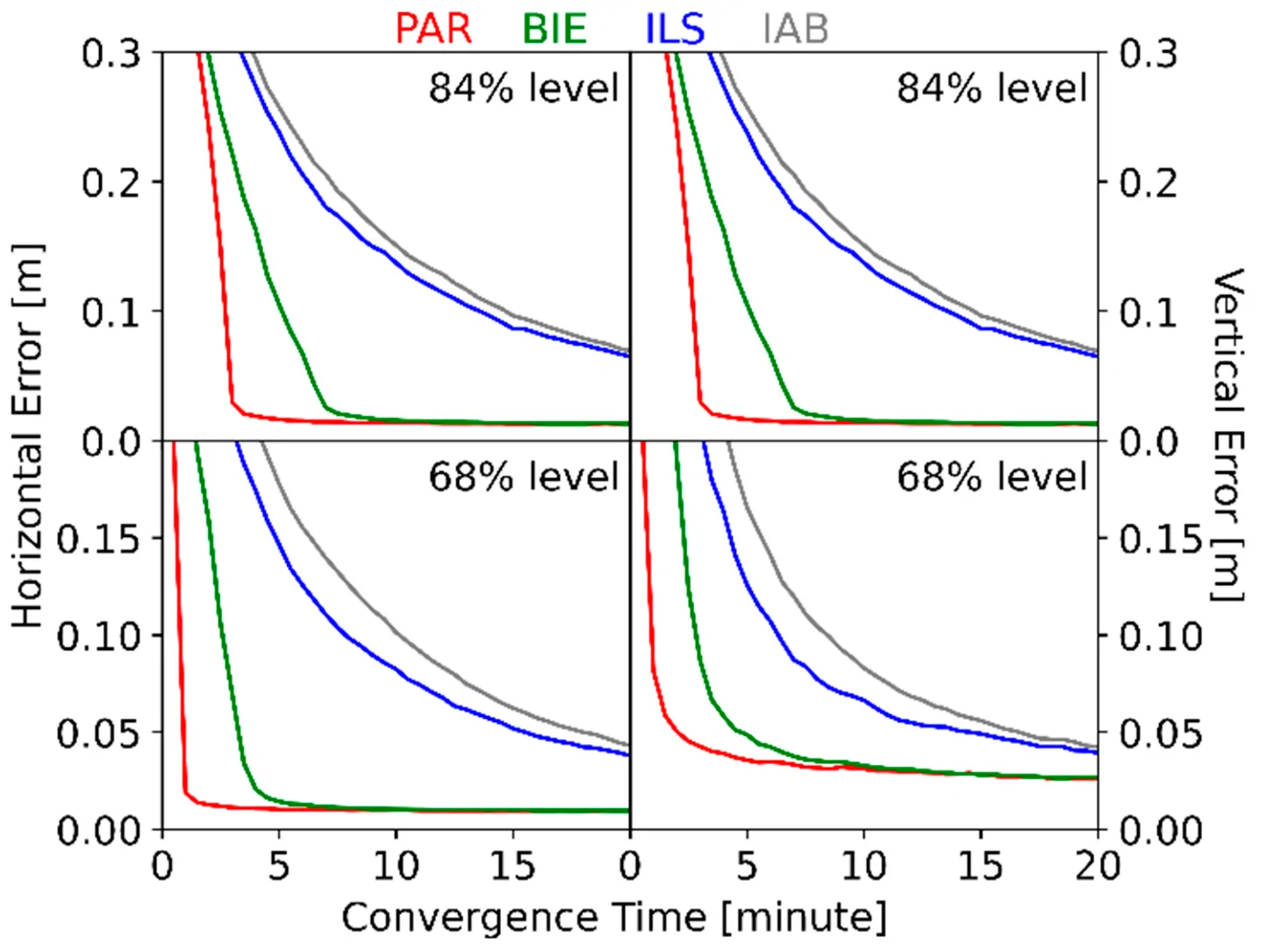
PPP convergence performance in different AR methods.

Figure 7 shows the horizontal and vertical errors of different AR methods under the triple-frequency condition. Table 3 presents the corresponding convergence time in the horizontal and vertical components, where convergence is defined as the epoch after which the horizontal and vertical positioning errors remain continuously below 0.1 m for 5 min. Convergence times are summarized at the 68th and 84th percentiles rather than by the mean, as the empirical distributions are positively skewed. The 84th percentile approximates the one-sided ±1*σ* level (84.13%) of a Gaussian distribution, while the 68th percentile follows the ±1*σ* two-sided coverage convention (68.27%) commonly used in GNSS accuracy reporting.

The results indicate that PAR achieves the fastest convergence among all strategies. At the 68th percentile, PAR converges within 1.0 min in both the horizontal and vertical directions. When the confidence level increases to the 84th percentile, the convergence time extends to 3.0 min, but PAR still outperforms the other methods by a large margin. This improvement is attributed to PAR’s selective exclusion of unreliable ambiguities and the improved reliability of the chosen subset, and thus together they support the rapid stabilization of the position solution.

In contrast, the BIE method shows moderately slower convergence, requiring 3.0 min (68%) and 5.5 min (84%). This is expected because BIE smooths the ambiguity space without committing to specific integer values, resulting in steadier but less decisive convergence behavior.

The ILS and IAB methods exhibit the slowest convergence. At the 68th percentile, ILS requires 8.0 and 6.5 min for horizontal and vertical convergence, respectively, while IAB requires 10.5 and 8.5 min, respectively. Under the stricter 84% criterion, convergence extends to 12–15 min. These delays may be attributed to their lower fixing rate and longer TTFF, which limit the availability of reliable fixed ambiguities during initialization and slow down the stabilization of the coordinate solution.

**Figure 8:**
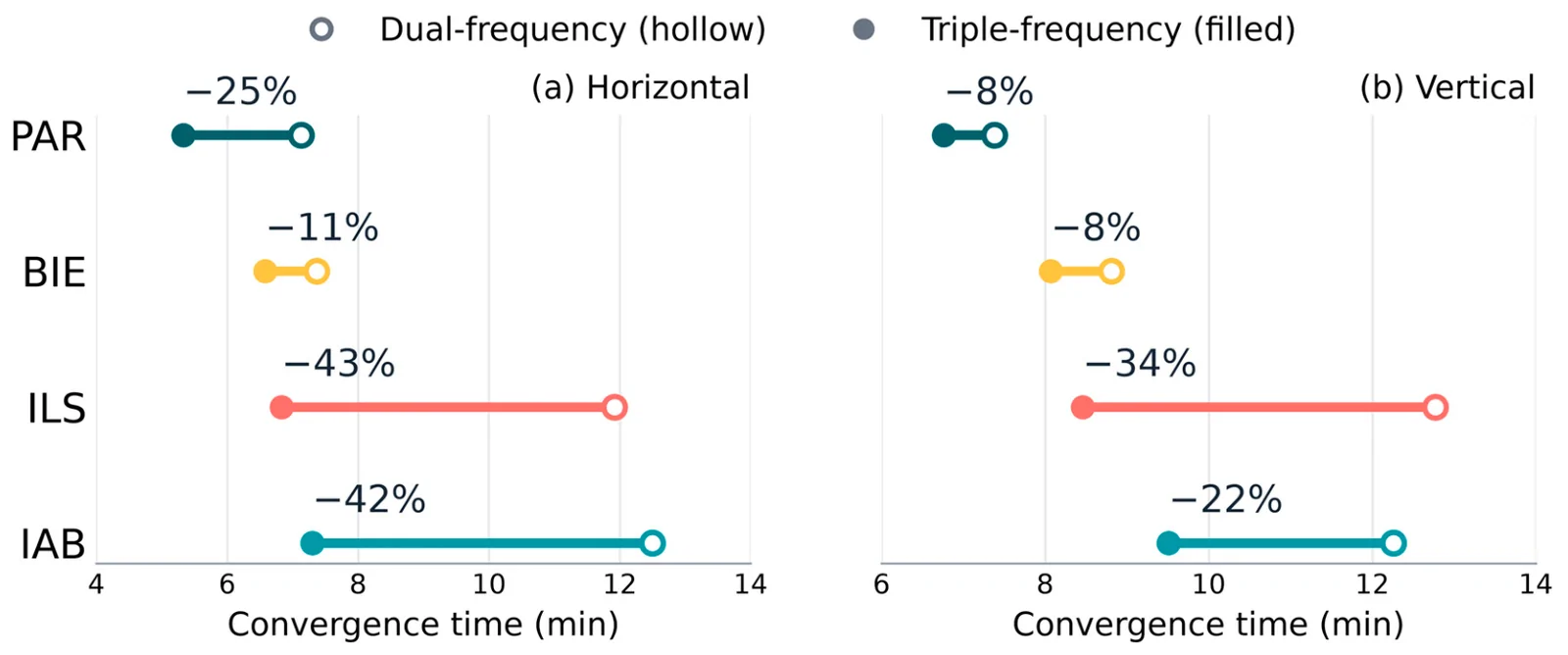
A comparison of the station-averaged 86th-percentile convergence time between dual-frequency (hollow) and triple-frequency (filled) PPP-AR for the four AR methods.

To separate the contribution of the third frequency from that of the AR methods themselves, the MGEX dataset was also re-processed in dual-frequency mode (GPS L1/L2, Galileo E1/E5a and BDS B1I/B3I, with WL/N1 cascading) under an otherwise identical processing strategy. For this comparison, the convergence time of each station is first summarized by the 86th percentile of all its re-convergence episodes across the 7 days, and the reported statistic is the mean of these per-station values over the 275 stations; this statistic complements the percentile measures in Table 3 by weighting every station equally while remaining robust to a station’s individual outlier episodes. The two modes are compared in Figure 8 and summarized in Table 4. The third frequency shortens this station-averaged 86th-percentile convergence time for all four methods but to clearly different degrees: ILS and IAB benefit the most (−43% and −42% horizontally), followed by PAR (−25%) and BIE (−11%). Vertically, the pattern is similar; ILS and IAB benefit the most. The large gains of ILS and IAB reflect the removal of their slow-converging tail: under dual frequency, a fraction of their sessions fail the acceptance test and remain in a less precise float state for an extended period, whereas the third frequency provides a substantially more precise float solution that ensures convergence even when integer commitment is not accepted.

#### 4.2.3. Positioning Accuracy

**Figure 9:**
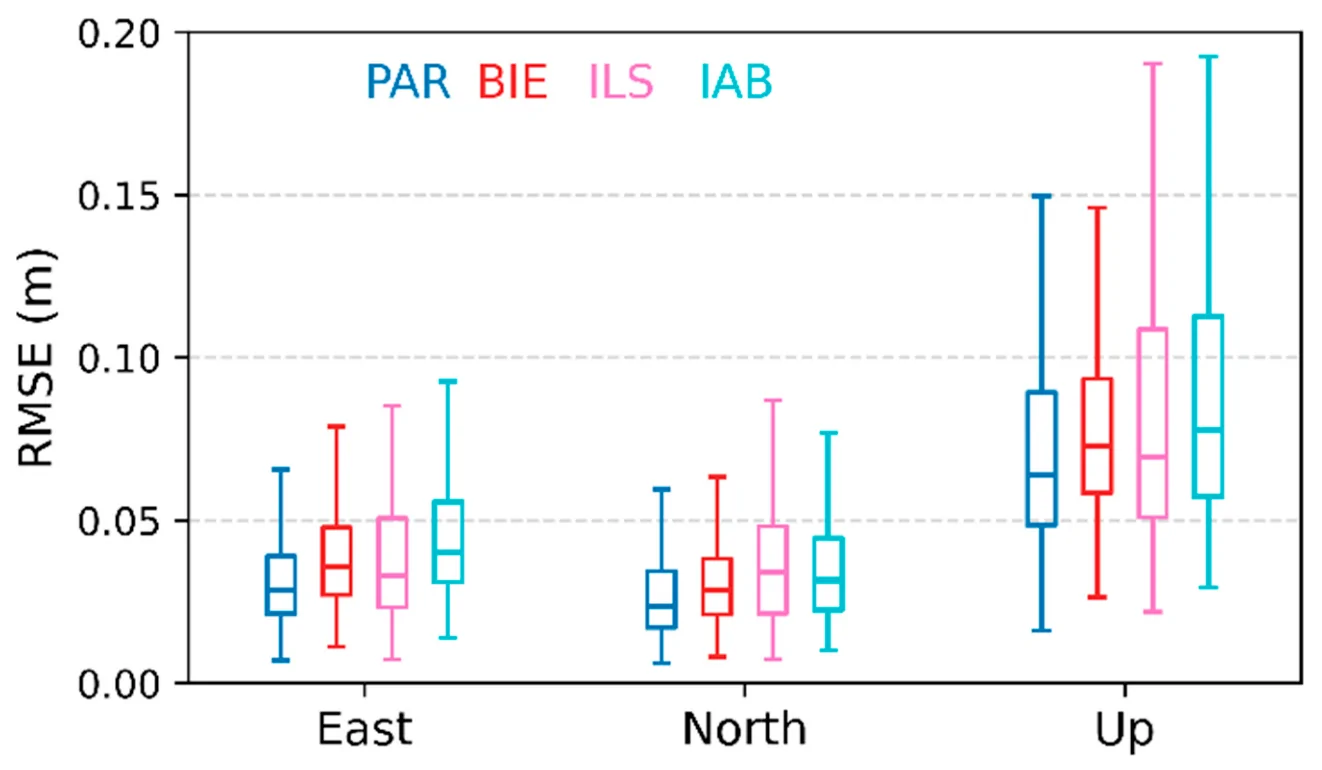
Positioning accuracy distribution of different AR methods.

Figure 9 illustrates the positioning precision distribution of different AR methods in the east, north, and up directions. The corresponding median positioning errors of the dual- and triple-frequency modes are summarized in Table 5.

PAR achieves the highest precision across all components, with median errors of 0.029 m (east), 0.023 m (north), and 0.064 m (up). These results represent a 19% improvement in horizontal RMS and a 12% improvement in vertical RMS compared with ILS, attributable to the method’s rapid TTFF and consistently high fixing rate, which provide accurate ambiguity estimation results.

In contrast, the ILS and IAB methods display reduced precision due to their relatively low fix rates and greater vulnerability to incorrect fixes under triple-frequency observations.

**Figure 10:**
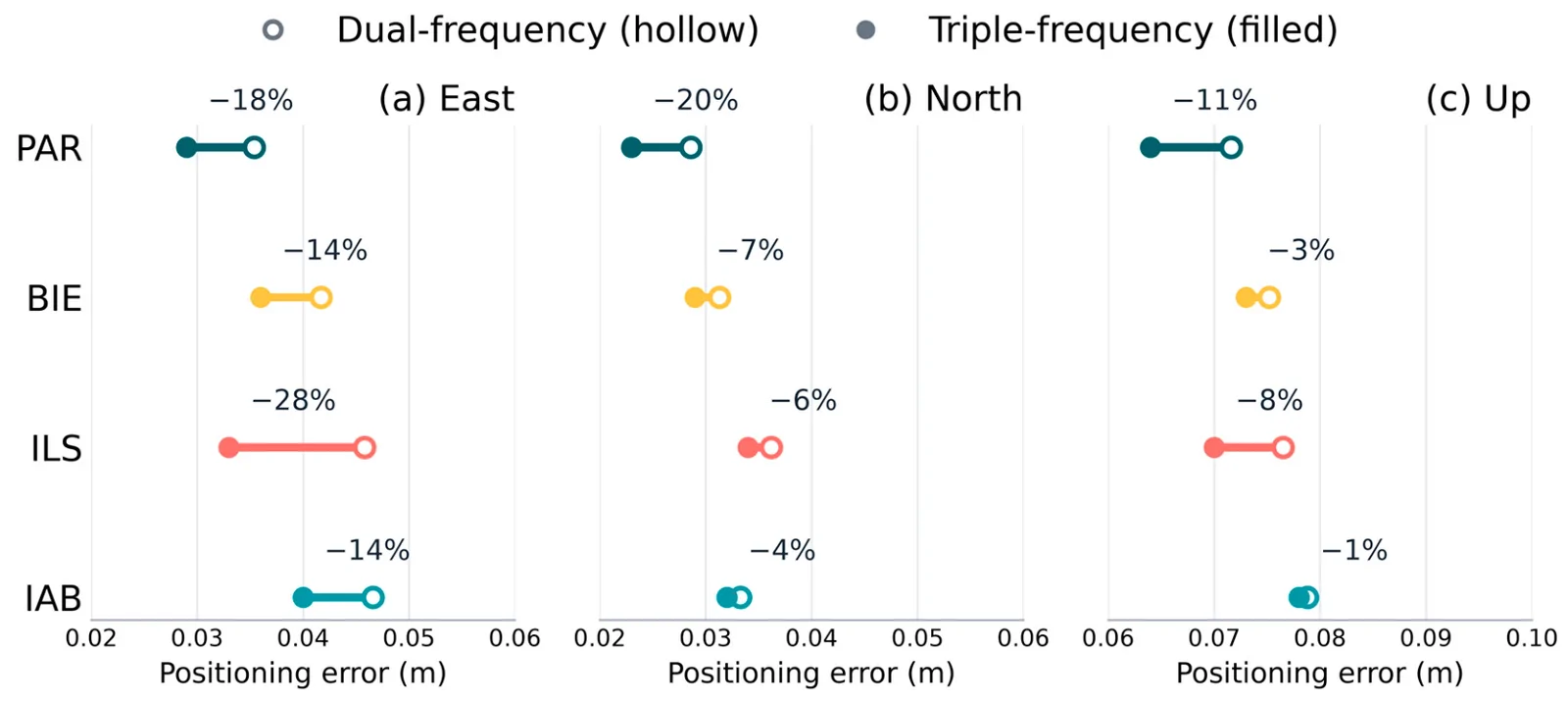
A comparison of dual- and triple-frequency median positioning errors for the four AR methods.

The dual-frequency counterpart of the median positioning errors is compared with the triple-frequency values in Figure 10. The third frequency improves the precision of all four methods in all three components, albeit modestly, with relative gains between 1% and 28%. The largest improvements occur for ILS in the east component (−28%, from 0.046 to 0.033 m) and for PAR in the north and up components (−20% and −11%). PAR remains the most precise method in both frequency modes, and the relative ordering of the four methods is largely preserved, indicating that the third frequency improves the positioning precision without fundamentally changing the comparative advantages of the four estimators.

#### 4.2.4. Exploring Which Satellite Would Be Excluded

PAR employs a minimum ratio exclusion strategy to progressively remove unreliable satellites. However, the ratio value, as a purely statistical test metric, lacks a direct and clear physical interpretation in terms of specific observation errors or satellite geometry. A natural question then arises: which satellites would be excluded during ambiguity resolution? To investigate this issue, ambiguity resolution (AR) information from DOY 100 of 2025 was analyzed. The ratio threshold was set to 1.8, keeping it consistent with the previous experiment.

Statistical analyses are conducted on the fractional parts of NL ambiguities (AmbFrac) in the NL fixing stage, which contains more noise, as well as on the satellite elevation angles involved in both WL and NL ambiguity fixing.

**Figure 11:**
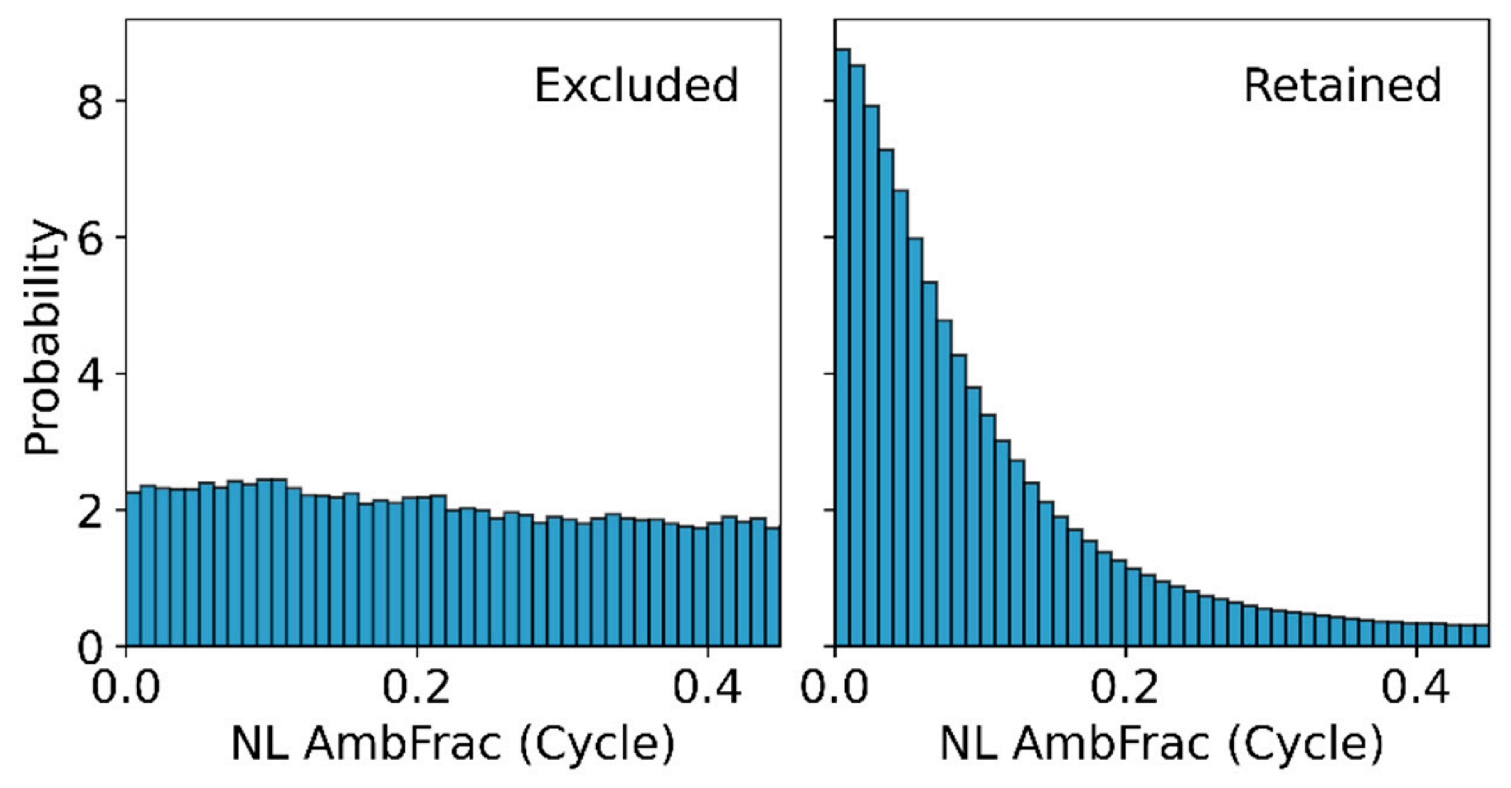
Distribution of NL AmbFrac for excluded (**left**) and retained (**right**) satellites.

The statistical results of NL AmbFrac are shown in Figure 11. It can be observed that the AmbFrac values of excluded satellites do not exhibit a sharp concentration near the half-integer boundary. Instead, compared with retained satellites, excluded satellites are more frequently associated with smaller AmbFrac values. This indicates that satellite exclusion in the NL fixing stage is not directly driven by large fractional ambiguity values but rather reflects the combined influence of ambiguity geometry and observation noise.

**Figure 12:**
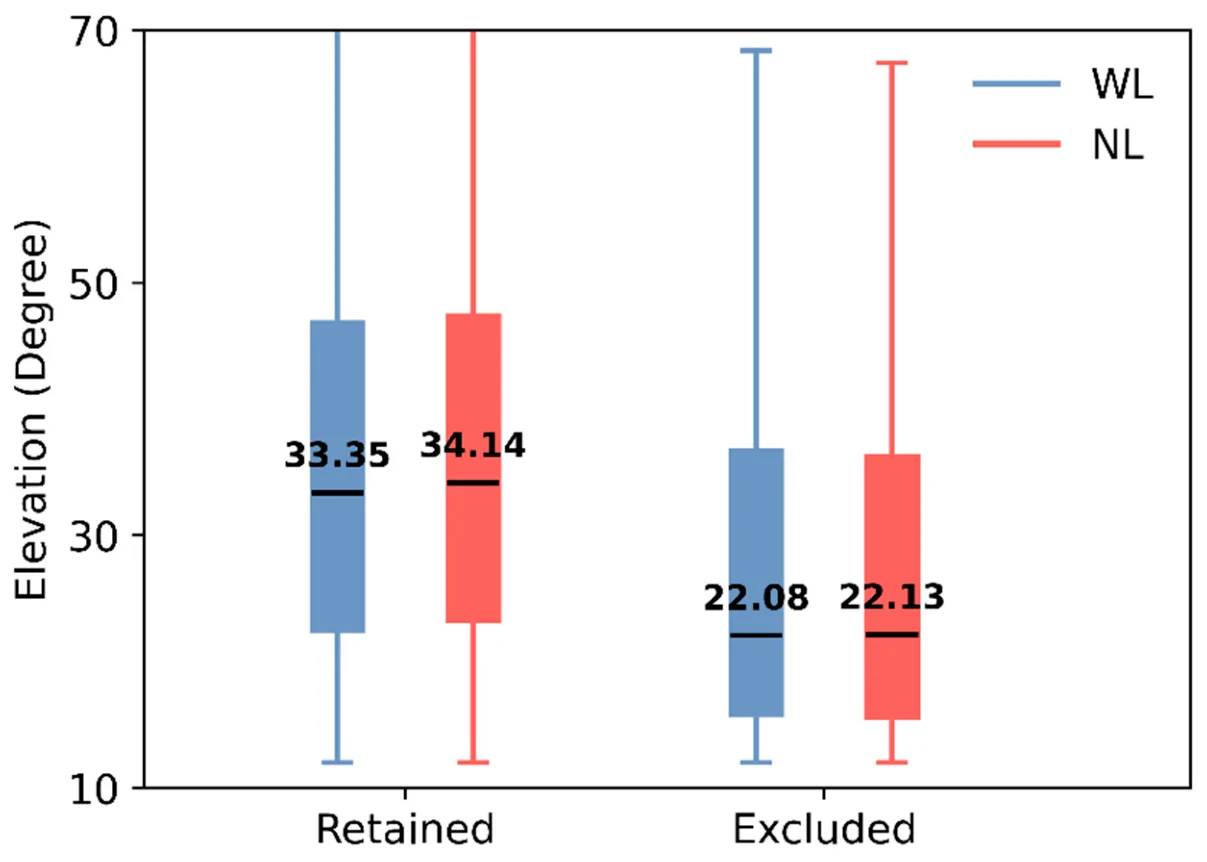
Distribution of elevation for excluded and retained satellites in WL and NL ambiguity fixed stage.

Figure 12 presents the elevation angle distributions of retained and excluded satellites during WL and NL ambiguity fixing. From the boxplots, the elevation angle distributions in the WL and NL stages exhibit nearly identical interquartile ranges and overall spreads. The median elevation of retained satellites is approximately 10–12° higher than that of excluded satellites. This behavior mainly reflects the intrinsic non-uniform distribution of satellite elevation angles rather than the selective exclusion of high-elevation satellites.

#### 4.2.5. Vehicle Kinematic PPP Test

**Figure 13:**
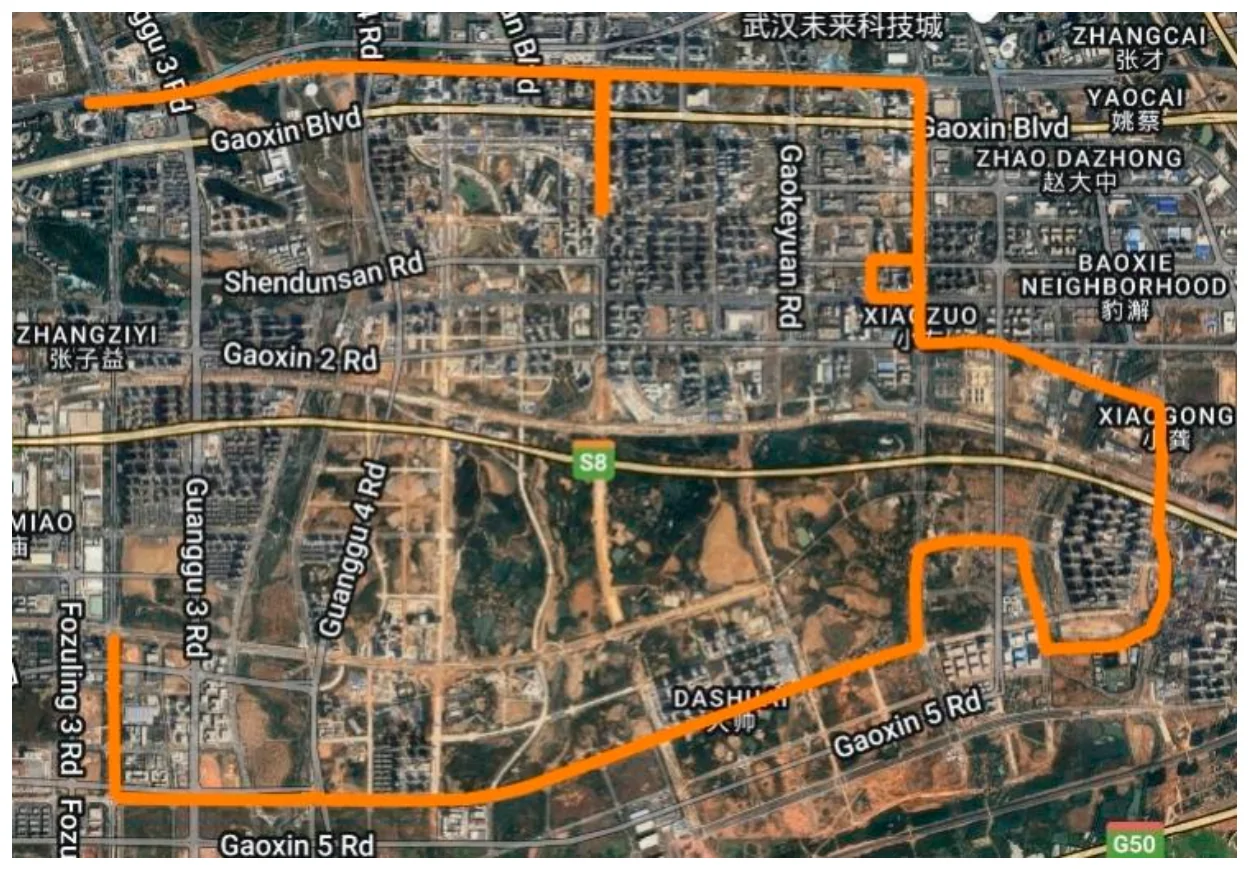
The trajectory of the vehicle. Base map labels are in Chinese; key place names are translated.

To evaluate the performance of different AR methods in a kinematic situation, we conducted a vehicle kinematic PPP experiment using the RTK test dataset provided by GREAT-PVT software, version 0.1.0. The data were collected at a sampling rate of 1 Hz by a Septentrio AsteRx4 receiver in Donghu High-Tech Zone, Wuhan, China, on DOY 351 of 2020. The trajectory of the vehicle is illustrated in Figure 13. The ambiguity resolution parameters were kept identical to those used in the static and simulated kinematic experiments.

**Figure 14:**
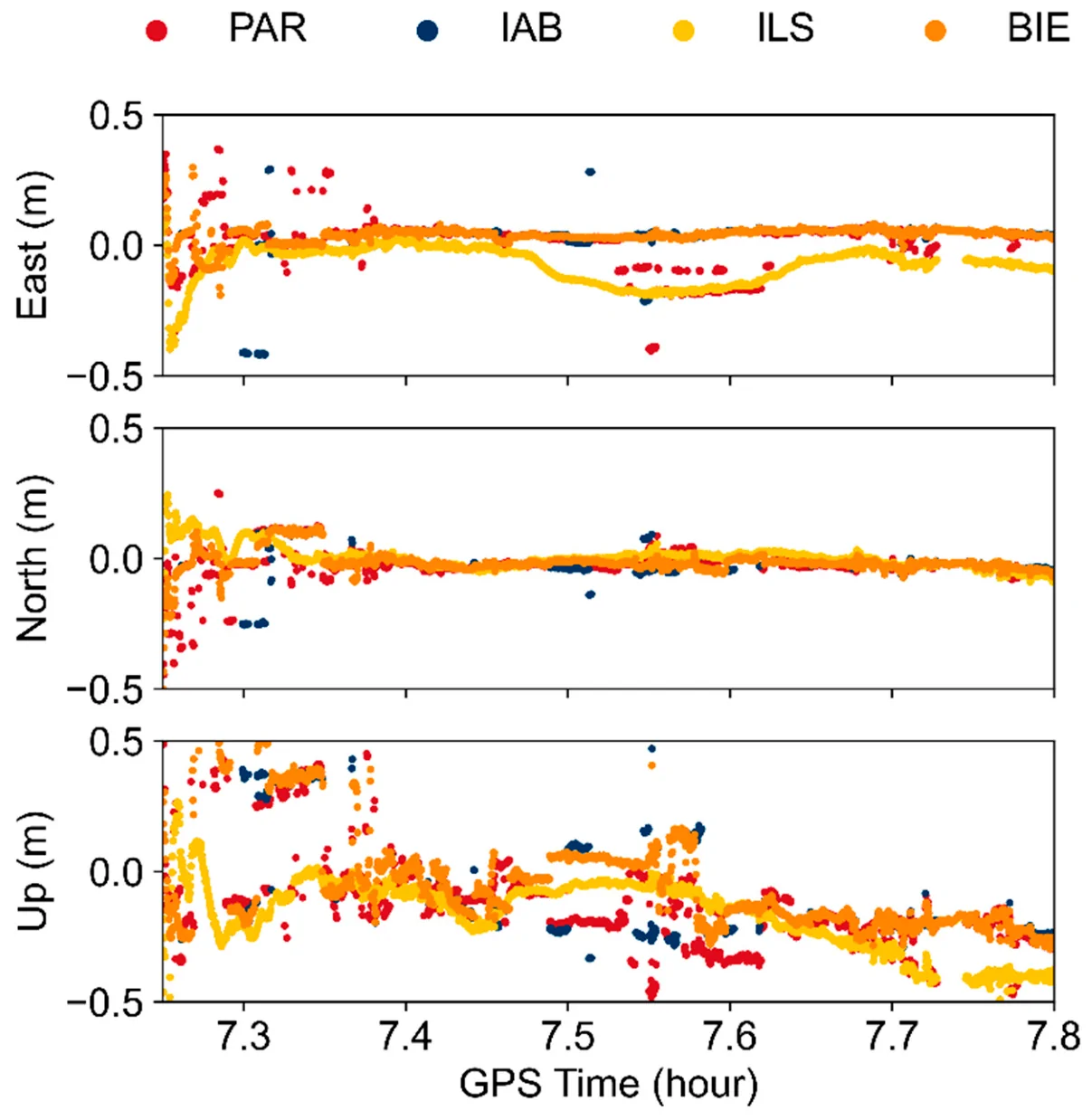
Positioning errors of different AR methods with vehicle observation (DOY 351/2020).

The reference coordinates for assessing PPP accuracy were derived from an RTK solution processed by GREAT-PVT software. Figure 14 displays the time series of positioning errors in the east, north, and up directions derived from the vehicle kinematic dataset using different AR methods. It is evident that the BIE estimator yields significantly more stable positioning errors in both the east and north components compared to others, with the smallest RMS errors of 0.043 m in the east and 0.059 m in the north components. Specifically, between GPS Time 7.5 and 7.7 h, several alternative methods exhibit significant deviations in the east component, underscoring the effectiveness of the BIE method in mitigating outliers and maintaining solution stability.

It should be noted that the vehicle test was conducted under predominantly open-sky conditions with only moderate dynamics and limited signal blockage or multipath conditions. The reported fixing rates and positioning accuracy should therefore be regarded as an optimistic baseline rather than worst-case performance. The up component exhibited slower convergence and greater fluctuations for all AR methods, which are attributable to the relatively weak vertical geometry (VDOP) during the initial phase of the session and the inherently slower vertical convergence of PPP-AR, rather than to signal blockage.

A comparative analysis of the ILS and PAR methods reveals a substantial difference in performance under the same minimum ratio threshold of 1.8. In kinematic scenarios, cycle slips and increased ambiguity variances severely degrade the reliability of fixing. Consequently, as summarized in Table 6, the ILS method achieves a very low fix rate of only 5.1%, and its positioning accuracy is notably inferior to that of PAR. In contrast, PAR maintains a high fix rate of 97.5%, demonstrating its practical advantage in maintaining reliable ambiguity resolution under dynamic conditions.

## 5. Summary and Conclusions

This study presents a comparative evaluation of four AR methods, ILS, PAR, BIE and IAB, in triple-frequency PPP-AR mode using a large MGEX dataset. The results quantify the trade-offs: PAR achieves the fastest convergence (1.0 min at 68th percentile) but incurs approximately 38 times higher computational cost than ILS; BIE provides superior kinematic stability (east RMS 0.043 m) but does not produce integer fixes; ILS fails dramatically under mild dynamics (fix rate 5.1%); and IAB suppresses wrong fixes at the cost of longer convergence. These findings offer practical guidance for method selection in real-world PPP applications. The main conclusions are as follows:

1. PAR achieves the best overall PPP-AR performance under realistic triple-frequency conditions. In static and kinematic tests, PAR provides the fastest convergence and highest fixing reliability. It attains sub-decimeter accuracy within about one minute for 68% of sessions and maintains a median fixing rate above 99.8% across 275 MGEX stations. This advantage stems from its minimum ratio-based satellite exclusion mechanism, which selectively removes unreliable ambiguities and ensures robust estimation even under difficult ionospheric and multipath conditions. Although computationally more expensive than other methods, the rapid convergence and superior precision justify this overhead in most practical applications. Further analysis reveals that excluded satellites predominantly have lower elevation angles (median 10–12° lower than retained ones) and are influenced more by overall ambiguity geometry and noise than by large fractional parts close to half-cycles.
2. BIE offers the highest positioning stability in kinematic conditions. Owing to its minimum-MSE optimality and equivariant smoothing of multiple integer candidates, BIE significantly reduces outlier influence and trajectory jumps. In the vehicular experiment, it produces the smallest horizontal RMS errors (0.043 m in the east and 0.059 m in the north), outperforming all methods. These results highlight the suitability of BIE for scenarios with rapidly changing satellite geometry, intermittent signal blockage, or mixed-quality measurements, where solution continuity and robustness are more critical than strict integer fixing.
3. The performance of ILS is highly sensitive to measurement quality and satellite geometry. Even in the MGEXs, its fixing rate distribution exhibits a long tail (5th percentile below 80%), and its TTFF reaches about 13 min at the 90th percentile. In the vehicular test, the fixing rate drops sharply to only 5.1% under the same ratio threshold of 1.8, far below that of IAB (77.4%) and PAR (97.5%).
4. IAB effectively suppresses incorrect fixes but at the cost of a reduced fixing rate and longer convergence time. The aperture-controlled pull-in region lowers the risk of erroneous integer commitments, but it also markedly decreases the fixing rate and prolongs the TTFF. Both static and kinematic experiments show that IAB converges more slowly and delivers lower positioning accuracy than PAR or BIE. Thus, IAB is the most appropriate for applications where avoiding wrong fixes is more important than rapid convergence or high availability.
5. A dual-frequency baseline reveals that in terms of the station-averaged 86th-percentile convergence time, the third frequency benefits all four methods but to clearly different degrees: ILS and IAB gain the most (−43% and −42% horizontally), mainly through the removal of the slow-converging tail of their dual-frequency solutions, whereas BIE gains the least (−11%). Its effect on positioning precision is more uniform and modest (1–28%), with PAR remaining the most precise method in both modes.

Among the methods evaluated, PAR proves the most effective for fast and accurate ambiguity resolution, while BIE provides strong stability in challenging kinematic environments. Future work could usefully explore hybrid or adaptive ambiguity resolution frameworks that integrate the strengths of multiple methods within a unified processing strategy, thereby enhancing overall robustness and adaptability.

## Figures and Tables

**Table 1:** Models and strategies for PPP-AR data processing.

Items	Descriptions
Estimator	Extended Kalman filter
Sampling interval	30 s
Elevation mask	7°
Combination	Undifferenced and uncombined
Antenna	Igs20.atx
Station coordinate	Estimated as kinematic
Troposphere model	Saastamoinen model
Cycle slip detects	MW method

**Table 2:** The mean computational time, std, and growth rate of the four AR methods.

AR Method	Mean (ms)	Std (ms)	Average Growth Rate
IAB	2.66	2.71	15.6%
ILS	4.54	20.14	20.3%
PAR	175.16	141.1	37.0%
BIE	211.14	304.12	27.6%

**Table 3:** Convergence time of different AR methods (unit: min).

AR Method	Horizontal (84%)	Vertical (84%)	Horizontal (68%)	Vertical (68%)
PAR	3.0	3.0	1.0	1.0
BIE	5.5	5.5	3.0	3.0
ILS	14.0	12.0	8.0	6.5
IAB	15.0	13.5	10.5	8.5

**Table 4:** The station-averaged 86th-percentile convergence time of the four AR methods in dual- and triple-frequency PPP (unit: min).

	PAR		BIE		ILS		IAB	
	H86	V86	H86	V86	H86	V86	H86	V86
triple-freq.	5.33	6.76	6.58	8.07	6.83	8.46	7.30	9.51
dual-freq.	7.13	7.38	7.37	8.81	11.92	12.77	12.50	12.26

**Table 5:** Median positioning errors of different AR methods in dual- and triple-frequency PPP (unit: m).

AR Method	East	North	Up
PAR	0.029	0.023	0.064
BIE	0.036	0.029	0.073
ILS	0.033	0.034	0.070
IAB	0.040	0.032	0.078
PAR (dual)	0.035	0.029	0.072
BIE (dual)	0.042	0.031	0.075
ILS (dual)	0.046	0.036	0.077
IAB (dual)	0.047	0.033	0.079

**Table 6:** Performance comparison of different AR methods.

AR Method	East RMS (m)	North RMS (m)	Up RMS (m)	Fix Rate (%)
PAR	0.088	0.100	0.308	97.531
BIE	0.043	0.059	0.363	-
ILS	0.105	0.085	0.330	5.137
IAB	0.100	0.092	0.334	77.424

## Data Availability

The original contributions presented in the study are included in article.

## References

[1] Ge M., Gendt G., Rothacher M.A., Shi C., Liu J. (2008). Resolution of GPS carrier-phase ambiguities in Precise Point Positioning (PPP) with daily observations. J. Geod..

[2] Xiang Y., Zhao Y., Tang T., Wang N., Li Z., Pei L., Yu W. (2025). Real-time extraction of multi-GNSS ionospheric observables based on PPP-AR. J. Geod..

[3] Lu C., Li X., Cheng J., Dick G., Ge M., Wickert J., Schuh H. (2018). Real-Time Tropospheric Delay Retrieval from Multi-GNSS PPP Ambiguity Resolution: Validation with Final Troposphere Products and a Numerical Weather Model. Remote Sens..

[4] Yang Y., Su K., Jiao G., Yan Z. (2026). Improved Global Ionospheric Modeling based on the Flexible combination between multi-GNSS PPP and Augmenting with IRI Virtual Stations. Adv. Space Res..

[5] Liu M., Tu R., Chen Q., Li Q., Chen J., Zhang P., Lu X. (2024). Study of high-precision time transfer method enhanced by PPP-AR/PPP-RTK. Meas. Sci. Technol..

[6] Bertiger W., Desai S.D., Haines B., Harvey N., Moore A.W., Owen S., Weiss J.P. (2010). Single receiver phase ambiguity resolution with GPS data. J. Geod..

[7] Teunissen P.J.G. Least-Squares Estimation of the Integer GPS Ambiguities. Invited Lecture, Section IV ‘Theory and Methodology’, General Meeting of the International Association of Geodesy, Beijing, China, August 1993. https://www.academia.edu/57679204/Least_squares_estimation_of_the_integer_GPS_ambiguities.

[8] Teunissen P.J.G. (2003). Towards a unified theory of GNSS ambiguity resolution. J. Glob. Position. Syst..

[9] Teunissen P.J.G. (1999). The probability distribution of the GPS baseline for a class of integer ambiguity estimators. J. Geod..

[10] Wen Z., Henkel P., Brack A., Günther C. (2012). Best Integer Equivariant estimation for Precise Point Positioning. Proceedings ELMAR-2012.

[11] Odolinski R., Teunissen P.J.G. (2020). Best integer equivariant estimation: Performance analysis using real data collected by low-cost, single- and dual-frequency, multi-GNSS receivers for short- to long-baseline RTK positioning. J. Geod..

[12] Massarweh L., Verhagen S., Teunissen P.J.G. (2024). New LAMBDA toolbox for mixed-integer models: Estimation and evaluation. GPS Solut..

[13] Laurichesse D., Blot A. Fast PPP Convergence Using Multi-constellation and Triple-frequency Ambiguity Resolution. Proceedings of the 29th International Technical Meeting of The Satellite Division of the Institute of Navigation (ION GNSS+ 2016).

[14] Geng J., Guo J., Meng X., Gao K. (2020). Speeding up PPP ambiguity resolution using triple-frequency GPS/BeiDou/Galileo/QZSS data. J. Geod..

[15] Abd Rabbou M., Abdelazeem M., Morsy S. (2021). Performance Evaluation of Triple-Frequency GPS/Galileo Techniques for Precise Static and Kinematic Applications. Sensors.

[16] Gazzino C., Lelarge N. A Cascading Approach for Multi-Frequency Widelanes and Extra-Widelanes Carrier Phase Integer Ambiguity Resolution. Proceedings of the 37th International Technical Meeting of the Satellite Division of The Institute of Navigation (ION GNSS+ 2024).

[17] Geng T., Wang X., Li Z., Li Z., Zhao Q. (2025). PPP ambiguity resolution for GPS/Galileo/BDS-3 using different multi-frequency OSB products. Adv. Space Res..

[18] Xiao G., Li P., Gao Y., Heck B. (2019). A Unified Model for Multi-Frequency PPP Ambiguity Resolution and Test Results with Galileo and BeiDou Triple-Frequency Observations. Remote Sens..

[19] Liu T., Chen Q., Geng T., Jiang W., Chen H., Zhang W. (2022). The benefit of Galileo E6 signals and their application in the real-time instantaneous decimeter-level precise point positioning with ambiguity resolution. Adv. Space Res..

[20] Zhang R., Tu R., Ge Y., Wang S., Lu X. (2025). An investigation into multi-frequency ambiguity resolution for real-time PPP time and frequency transfer. Meas. Sci. Technol..

[21] Geng J., Chen X., Pan Y., Mao S., Li C., Zhou J., Zhang K. (2019). PRIDE PPP-AR: An open-source software for GPS PPP ambiguity resolution. GPS Solut..

[22] Li X., Yuan L., Li X., Huang J., Shen Z., Tan Y. (2025). GREAT-PVT: An open-source software for multi-frequency and multi-GNSS PPP-AR and RTK. GPS Solut..

[23] Zhou F., Dong D., Ge M., Li P., Schuh H. (2017). Simultaneous estimation of GLONASS pseudorange inter-frequency biases in precise point positioning using undifferenced and uncombined observations. GPS Solut..

[24] Gu S., Lou Y., Shi C., Liu J. (2015). BeiDou phase bias estimation and its application in precise point positioning with triple-frequency observable. J. Geod..

[25] Deng J., Zhang A., Zhu N., Ke F. (2020). Extra-Wide Lane Ambiguity Resolution and Validation for a Single Epoch Based on the Triple-Frequency BeiDou Navigation Satellite System. Sensors.

[26] Teunissen P.J.G. (1995). The least-squares ambiguity decorrelation adjustment: A method for fast GPS integer ambiguity estimation. J. Geod..

[27] Teunissen P.J.G. (1999). An optimality property of the integer least-squares estimator. J. Geod..

[28] Teunissen P.J.G., Joosten P., Tiberius C. (1999). Geometry-free Ambiguity Success Rates in Case of Partial Fixing. Proceedings of the 1999 National Technical Meeting of the Institute of Navigation.

[29] Teunissen P.J.G., Verhagen S. (2009). The GNSS ambiguity ratio-test revisited: A better way of using it. Surv. Rev..

[30] Teunissen P.J.G. (2003). Theory of integer equivariant estimation with application to GNSS. J. Geod..

[31] Yang Y., Zhou F., Song S. (2023). Improving precise point positioning (PPP) performance with best integer equivariant (BIE) estimator. GPS Solut..

[32] Teunissen P.J.G. (2005). Integer aperture bootstrapping: A new GNSS ambiguity estimator with controllable fail-rate. J. Geod..

